# A portent of catastrophic carbon dioxide embolism in laparoscopic hepatectomy: A case report

**DOI:** 10.1097/MD.0000000000038468

**Published:** 2024-06-14

**Authors:** Mei Li, Bing Yan, Mi Wang, Shengmei Zhu, Xianhui Kang

**Affiliations:** a Department of Anesthesiology, the First Affiliated Hospital, Zhejiang University School of Medicine, Hangzhou, Zhejiang Province, China; b Department of Anesthesiology, Haining People’s Hospital, Haining, Zhejiang Province, China.

**Keywords:** Carbon dioxide embolism, Laparoscopic hepatectomy, End-tidal carbon dioxide, Arterial blood analysis

## Abstract

**Introduction:**

Laparoscopic hepatectomy (LH) poses a high risk of carbon dioxide embolism due to extensive hepatic transection, long surgery duration, and dissection of the large hepatic veins or vena cava.

**Patient concerns:**

A 65-year-old man was scheduled to undergo LH. Following intraperitoneal carbon dioxide (CO_2_) insufflation and hepatic portal occlusion, the patient developed severe hemodynamic collapse accompanied by a decrease in the pulse oxygen saturation (SpO_2_).

**Diagnosis:**

Although a decrease in end-tidal carbon dioxide (ETCO_2_) was not observed, CO_2_ embolism was still suspected because of the symptoms.

**Interventions and Outcomes:**

The patient was successfully resuscitated after the immediate discontinuation of CO_2_ insufflation and inotrope administration. CO_2_ embolism must always be suspected during laparoscopic surgery whenever sudden hemodynamic collapse associated with decreased pulse oxygen saturation occurs, regardless of whether ETCO_2_ changes. Instant arterial blood gas analysis is imperative, and a significant difference between PaCO_2_ and ETCO_2_ is indicative of carbon dioxide embolism.

**Conclusion:**

Instant arterial blood gas analysis is imperative, and a significant difference between PaCO_2_ and ETCO_2_ is indicative of carbon dioxide embolism.

## 1. Introduction

Laparoscopic hepatectomy (LH) is an increasingly popular surgical treatment option for patients with liver disease. Despite the many advantages of LH, it is a well-known complication of CO_2_ embolism. This complication, which is associated with a high mortality rate of approximately 28%,^[1]^ may result in blockage of blood flow through the right ventricle (RV) or pulmonary artery, which may further result in heart failure.^[2]^ LH poses a high risk of CO_2_ embolism due to extensive hepatic transection, long duration of the surgery, and dissection of the large hepatic veins or vena cava.

End-tidal carbon dioxide (ETCO_2_) monitoring has been suggested as a sensitive and non-invasive tool to monitor CO_2_ embolism; most reports show that a significant decrease in ETCO_2_ suggests CO_2_ embolism.^[3]^ In most studies, a marked decrease in ETCO2 suggests CO_2_ embolism, but some literature states that in CO_2_ embolization the drop in ETCO2 may be slight and transient or the ETCO2 drop may not be observed.^[4]^

We report the case of a patient who suffered CO_2_ embolism without decreased ETCO_2_ during LH. The patient survived after immediate desufflation, ventilation with 100% oxygen, hyperventilation, and inotropic support.

## 2. Case presentation

The patient was a 65-year-old man with a body mass index of 21 kg/m^2^ and American Society of Anesthesiologists physical status II. He was diagnosed with primary hepatic carcinoma and scheduled for LH in June 2018. The medical history revealed hypertension without normative treatment. His preoperative vital signs were as follows: blood pressure (BP) 180/70 mm Hg; and heart rate (HR), 68 beats per minute (bpm). Other examinations, including hepatic function tests, hematological and serum biochemical studies, electrocardiography, and chest radiography, detected no obvious abnormalities.

The patient was scheduled for surgery and transferred to the operating room. Preoxygenation and monitoring were performed, which showed a BP of 180/70 mmHg, HR of 65 bpm, and pulse oxygen saturation (SPO_2_) 100%. Considering his hypertension, 15 mg urapidil was administered instantly and dexmedetomidine (0.5µg/kg/h) was maintained for 40 minutes intravenously. Both the radial artery and internal jugular vein were cannulated under ultrasonic guidance to provide continuous monitoring of the arterial BP and central venous pressure, respectively. The BP decreased to 160/70 mm Hg 15 minutes later, after which general anesthesia was induced with 0.5 mg atropine, 12, 50, and 10 mg of etomidate, sufentanil, and cisatracurium, respectively. After tracheal intubation, continued mechanical ventilation of the lungs was initiated using a carbon dioxide absorption anesthetic breathing system. Anesthesia was maintained with continuous administration of propofol (4–10 mg/kg/h), remifentanil (0.1–0.2 µg/kg/min) and cisatracurium (0.1 mg/kg/h). The patient was then placed in a head-up and left-lateral position, and the operation was started.

Approximately 20 minutes later, the patient’s BP gradually declined to 80/50 mm Hg after blocking the hepatic portal. The pneumoperitoneal pressure was 12 mm Hg, and the CVP was 5 cmH_2_O, with bleeding of 200 ml, urine output of 160 ml, and infusion of approximately 2000 ml. Ephedrine (6 mg) was administered intravenously to improve BP. Because of unstable BP, ephedrine (6 mg) was administered again. However, approximately 15 minutes later, severe hypotension abruptly manifested with a decrease in BP from 120/70 mm Hg to 75/50 mm Hg. The decrease in BP was also accompanied by hypoxemia, involving a decrease in SpO_2_ from 100% to 85%. As the ETCO_2_ was in the range of 36 to 43 mm Hg, the resident did not initially consider pulmonary embolism initially. In order to maintain hemodynamic stability, adrenaline (100µg) was administered repeatedly and phenylephrine (0.1–2 µg/kg/min) was also continuously infused.

Instant arterial blood gas analysis was performed simultaneously, which showed the following levels (the fraction of inspired oxygen was 60%): pH 7.07, PaCO_2_ 101 mm Hg, and PaO_2_ 77 mm Hg. Combining all the clues, the most probable reason for hemodynamic collapse and hypoxemia in this case was CO_2_ embolism. We had taken a number of measures, including stopping surgical insufflation, adopting ventilation with 100% oxygen, hyperventilation, increasing positive end-expiratory pressure, infusing epinephrine and phenylephrine, and speeding up infusion. Systemic BP was maintained above 100 mm Hg with inotrope support, and 100% oxygen was administered, although systemic BP dropped from 110 mmHg to 80 mm Hg for several minutes.

After 10 minutes, a stable hemodynamic condition was achieved; the BP was in the range of 90/50 mm Hg to 150/90 mm Hg. In addition, SpO_2_ was 100% again. A total of 100 µg epinephrine was administered, and approximately 3 mg phenylephrine was continuously infused, and the infusion was further accelerated thereafter. The surgery lasted for 135 minutes, with a total infusion volume of 3500 ml, bleeding volume of 400 ml, and urine output of 450 ml. Before leaving the operating room, atrial blood gas analysis was performed again, which showed a pH of 7.32, PaCO_2_ 46.5 mm Hg, and PaO_2_ 162 mm Hg. The patient was transferred to the post-anesthesia care unit (PACU) for further observation. As his condition improved and vital signs remained stable, the patient was extubated in the PACU without any complications.

## 3. Discussion

In recent years, LH has been regarded as an effective and safe surgical procedure for liver disease in select patients. However, laparoscopic surgery could result in CO_2_ embolism.^[5]^ Additionally, the unique hepatic anatomy, which has numerous large veins and the vena cava, can allow gas to enter into the circulatory system through high-pressure insufflation if vessel injuries occur during dissection of the extensive liver parenchyma.^[6]^ As large gas emboli resulting in serious complications during laparoscopic liver biopsy are reported to occur in only 0.0016% of cases, anesthetists and surgeons might have underestimated the incidence of CO_2_ embolism before.^[7]^ Kim et al^[8]^ reported the incidence of CO_2_ embolism during total LH using transesophageal echocardiography (TEE) and stated that gas embolism was observed in all patients.

CO_2_ emboli can obstruct the right chamber of the heart and pulmonary artery, which is a potentially fatal complication of anesthesia administered during laparoscopic surgery. The meaningful phenomena of gas embolism include decreased ETCO_2_, hypoxemia, hypercapnia, high airway pressure, and hemodynamic changes. In this case, the initial presentation was hypotension and hypoxia without decreased ETCO_2_. Therefore, we suspected other diseases such as hemorrhage, heart failure, and pneumothorax. Hepatic portal occlusions can also cause hypotension. However, the estimated blood loss was 200 ml, with 160 ml urine output and 2000 ml fluid infusion. Moreover, instant auscultation confirmed the presence of bilateral breath sounds. Considering the symptoms of hypoxia, rapid arterial blood analysis was performed. The diagnosis of CO_2_ embolism was confirmed on the basis of the significant difference between PaCO_2_ and ETCO_2._

This case demonstrates that severe hypotension accompanied by hypoxemia may indicate CO_2_ embolism during laparoscopic surgery, regardless of ETCO_2_ decreases. Unlike air or oxygen, CO_2_ is more soluble in blood. Therefore, the clinical impact of CO_2_ embolism during laparoscopy might be limited, and acute hypoxemia is rare. In addition, the pneumoperitoneum interferes with the potential hemodynamic effects due to CO_2_ embolism, complicating the clinical diagnosis.^[9]^ ETCO_2_ monitoring is not very specific, and it may increase initially.^[10]^ Moreover, the changes in ETCO_2_ are rapid and sometimes of short duration; they could easily be missed in the operating theater.^[11]^

Surgeons should be informed immediately and stop insufflation when there is clinical suspicion of CO_2_ embolism.^[12]^ Ventilation with 100% oxygen could be used to wash out CO_2_ and reduce ventilation-perfusion mismatch and hypoxemia.^[13]^ Hyperventilation is also used to help eliminate CO_2_. Additionally, supportive treatment with fluids, vasopressors, and cardiopulmonary bypass may be necessary for patients with severe cardiovascular collapse.^[8]^ Meanwhile, instant arterial blood analysis is especially important, which helps to diagnose the significant difference between PaCO_2_ and ETCO_2_.

Besides ETCO_2_ monitoring, TEE is the most sensitive method to detect CO_2_ embolization compared with ETCO_2_ and mean pulmonary artery pressure changes.^[14]^ TEE can detect intravenous injection of CO_2_ as small as 0.02 ml/kg.^[15]^ However, the process of CO_2_ embolism usually progresses rapidly. Thus, knowledge of the clinical symptoms of CO_2_ embolism is much more practical. Furthermore, a central venous pressure line should be inserted in cardiac patients or in hemodynamically unstable patients, not only for hemodynamic monitoring but also as a way of attempting to remove gas emboli from the right atrium whenever gas embolization is suspected.^[16]^

## 4. Conclusion

This case shows that severe hypotension and hypoxemia under general anesthesia may indicate carbon dioxide embolism, although ETCO_2_ does not decrease. Instant arterial blood gas analysis is imperative, and a significant difference between PaCO_2_ and ETCO_2_ is indicative of carbon dioxide embolism. If carbon dioxide embolism occurs, prompt diagnosis and correct treatment are essential to improve the prognosis.

## Author contributions

**Supervision:** Shengmei Zhu.

**Writing – original draft:** Mi Wang, Mei Li.

**Writing – review & editing:** Xianhui Kang, Bing Yan.
